# Asthma and Vitamin D Deficiency: Occurrence, Immune Mechanisms, and New Perspectives

**DOI:** 10.1155/2022/6735900

**Published:** 2022-07-15

**Authors:** Fardis Salmanpour, Naghmeh Kian, Noosha Samieefar, Mohammad Amin Khazeei Tabari, Nima Rezaei

**Affiliations:** ^1^Student Research Committee, School of Medicine, Ahvaz Jundishapur University of Medical Sciences, Ahvaz, Iran; ^2^Network of Interdisciplinarity in Neonates and Infants (NINI), Universal Scientific Education and Research Network (USERN), Tehran, Iran; ^3^USERN Office, Shahid Beheshti University of Medical Sciences, Tehran, Iran; ^4^Student Research Committee, School of Medicine, Shahid Beheshti University of Medical Sciences, Tehran, Iran; ^5^Mazandaran University of Medical Sciences, Sari, Iran; ^6^Student Research Committee, School of Medicine, Mazandaran University of Medical Sciences, Sari, Iran; ^7^Research Center for Immunodeficiencies, Children's Medical Center, Tehran University of Medical Sciences, Tehran, Iran; ^8^Department of Immunology, School of Medicine, Tehran University of Medical Sciences, Tehran, Iran

## Abstract

Asthma, as a chronic inflammatory condition of the airways, has a considerable prevalence among children. Vitamin D might play a role in asthma pathogenesis by affecting the development of the lung, regulating the immune responses, and remodeling of airway smooth muscle (ASM). Study results on the association between the serum level of vitamin D and asthma severity have suggested a converse relationship between lower vitamin D levels and more severe clinical courses. However, they are not consistent in these findings and have shown insignificant correlations, as well. The possible effects of vitamin D on asthma have led researchers to consider this vitamin a potential prophylactic and therapeutic tool for managing children with variant degrees of asthma. Adding vitamin D to the routine corticosteroid therapy of asthmatic children is another field of interest that has shown promising results. In this narrative review study, we aim to elaborate on the existing knowledge on the role of vitamin D in asthma pathogenesis and prognosis, explain the controversies that exist on the effectiveness of treating patients with vitamin D supplements, and make a general conclusion about how vitamin D actually is linked to asthma in children.

## 1. Introduction

Recent studies are on their way to find the link between vitamin D deficiency and asthma in children [1, 2]. Asthma is one of the most common chronic diseases among children [3], affecting approximately 300 million people in the last few decades, and has already prevailed worldwide [4]. A combination of genetic susceptibility, host factors, and environmental exposures has roles in asthma pathogenesis [5]. Narrowing of the airway causes asthma and its symptoms [4].

The roles of vitamin D in the regulation of metabolism and calcium-phosphorus absorption of bone are among the known roles of this vitamin. However, the presence of vitamin D receptor (VDR) in other different organs and tissues shows that vitamin D physiology is not limited to mineral homeostasis and skeletal health maintenance [6]. Vitamin D could play effective roles in other systems, particularly, the immune system, and can help in the suppression of certain autoimmune diseases [7]. Our body receives vitamin D from a few number of foods, but the majority of vitamin D is produced through dermal synthesis [8].

Recent studies have reported an association between vitamin D deficiency and increased risk of asthma occurrence and exacerbation [2, 9–11]. Furthermore, in line with these findings, a study provided evidence about the association between vitamin D levels and the presence of asthma markers (i.e. immunoglobulin (Ig) E and eosinophil) [12]. Vitamin D might play a role in asthma pathogenesis by affecting the development of the lungs, regulating the immune responses and remodeling of airway smooth muscle (ASM) [13]. The lower levels might be associated with severe forms and more severe manifestations [14].

Several studies have failed to find significant changes in the asthma status of patients treated with vitamin D supplementation [2, 11, 15]. However, there were some limitations affecting the results, including the rate of severe asthma exacerbations in the placebo group being even lower than expectation [16]. The findings could not be generalized for all ages or situations, since is difficult to monitor the use of vitamin D supplementation [16]. On the other hand, insufficient vitamin D supplementation dose may cause treatment by vitamin D supplementation to fail and not affect the exacerbation rates, as expected [17].

This study is aimed at reviewing the recent researches on the effect of vitamin D deficiency on asthma pathogenesis, using vitamin D3 supplementation as prevention in the prenatal period or by adding on corticosteroid therapy.

### 1.1. A Brief Introduction to Asthma

Asthma is a chronic inflammatory disorder that affects the respiratory system. Hyperresponsiveness of the airways causes asthma symptoms that are wheezing, dyspnea, chest tightness, and cough, mainly at night or early in the morning [4]. Asthma phenotypes depend on the nature of the trigger (e.g., allergic vs. nonallergic), the age of onset (preschoolers vs. school-children vs. late-onset), and the pathophysiology involved (high vs. low T helper (Th) 2 cell-mediated responses, eosinophilic vs. noneosinophilic). Asthma clinical presentation is not constant, and the manifestations may be reduced or changed to the more severe symptoms and cause irreversible obstructive changes in the lungs [13].

Asthma is a highly complex, immune-mediated, inflammatory disease, with intermittent and reversible lower airways obstruction due to smooth muscle constriction and airway narrowing in response to environmental triggers, often in association with a viral upper respiratory tract infection. Numerous inflammatory pathways causing airway swelling contribute to the many clinical phenotypes of pediatric asthma [18].

Narrowing of airways causes asthma and its symptoms. In response to multiple bronchoconstrictor, mediators, and neurotransmitters, the airway smooth muscles get contracted and this leads to narrowing of the airway; this can be retreated by bronchodilators [4].

Cytokine-mediated inflammation in allergic asthma is the most important recent discovery in asthma pathogenesis. The airways of a sensitized child with asthma release mediators, damaging the epithelium. Th2 lymphocytes trigger the release of cytokines (interleukin- (IL-) 4, IL-5, IL-9, IL-13, among others), which upregulate or downregulate other cytokine receptors. Cytokines stimulate IgE production, mast cells, basophils, and eosinophils, which mediate inflammation via histamine, prostaglandins, and leukotrienes. Newer therapies interrupt the inflammatory pathway by inhibiting the actions of specific mediators (IgE and IL-5) [18].

Various factors can affect asthma pathogenesis and cause asthma exacerbation in early childhood. Some researches represented two types of factors that have role in asthma, host and environmental factors. Host factors contain genetics (asthmatic parents) and gender (prevalence of asthma is more common among boys). Environmental factors include allergen, infection, tobacco, smoke, outdoor/indoor pollutants, and diet. Some studies have shown that some environmental stimulus have effects on asthma pathogenesis such as maternal infection and smoking during pregnancy [4, 19].

### 1.2. Vitamin D Deficiency in Asthmatic Children

#### 1.2.1. Vitamin D Metabolism and Physiology

Our body receives vitamin D from a few number of foods but the majority of vitamin D is produced through dermal synthesis [8, 9, 20]. In the skin, 7-dehydrocholesterol (provitamin D3) is photolyased by UV-B radiation and converted to previtamin D3 (1 alpha, 25-dihydroxy) [6]; pro-vitamin D3 (from skin and diet) is then converted to 1,25-dihydroxyvitamin D3 (1,25(OH)2D3)—biologically active form 1,25-dihydroxyvitamin D—in two sequential hydroxylation steps: first in the liver (to become 25(OH)D3) and second in the kidneys.

The hormonal function of vitamin D is achieved through a single VDR which is a member of the class II steroid hormones and contains 427-amino acids [7]. VDR is present in different tissues and organs [6] and is responsible for a wide range of vitamin D functions. To name the major ones, vitamin D is best known for mineralizing the skeleton and increasing serum calcium and phosphorus concentrations; it plays effective roles in other systems, especially, the immune system, and can help in the suppression of certain autoimmune diseases [7].

#### 1.2.2. Vitamin D Deficiency

According to a study done in Tehran city of Iran, the 25(OH)D serum levels were proximity 116 nmol/l among the pediatric population [21]. According to available data, a low vitamin D level is common at any age, especially in girls and women [22]. Most studies have suggested 30 nmol/l as a cut-off point for defining vitamin D deficiency [22, 23]. The high-risk groups for vitamin D deficiency are children (especially those born with low weight), pregnant women, and elderlies [23]. Vitamin D deficiency is more common in adolescents living in Europe, the Middle East, and Asia [24–27]. This condition is significantly associated with aging—as a 70-year-old individual is thought to have 25% of the 7-dehydrocholesterol that a young adult has—and obesity—since vitamin D is fat-soluble [8].

Furthermore, a number of medications (like antiseizure drugs and glucocorticoids) and fat malabsorption could be the cause of vitamin D deficiency [8]. Low vitamin D level is also associated with disorders such as metabolic syndrome, cancers, and autoimmune, psychiatric, and neurodegenerative diseases [28].

#### 1.2.3. Is Vitamin D Deficiency Associated with Asthma Occurrence?

Recent studies are on their way to find the link between vitamin D and asthma in children [1, 2]. Studies recently have reported an association between vitamin D deficiency and increased risk of asthma [2, 9]. A case-control study was conducted to compare serum vitamin D levels between asthmatic children (*n* = 483) and healthy children (*n* = 483); the result showed that vitamin D deficiency was more common among asthmatic children [29]. Also, results showed that 68.1% of asthmatic children had deficiency in serum vitamin D levels (≤15 ng/ml) and 31.28% of them had insufficient levels (15 ng/ml< and >20 ng/ml) [29, 30]. In line with these findings, a study provided evidence about the association between vitamin D levels and the presence of asthma markers (i.e., IgE and eosinophil). The authors suggested that lower vitamin D levels correlate with increased total IgE amount and eosinophil count [12]. However, there are studies in contrast [31]. Notably, serum vitamin D level is considered one of the predictor factors for asthma which is even more accurate than serum IgE level and familial history of asthma [29]. The level of vitamin D in the serum was not in associated with the severity of asthma in some studies [32]. However, there have also been reports of correlation between the vitamin D level and asthma severity in contrast [14, 33].

Similarly, the results from another case-control study supported an association between vitamin D deficiency and increased risk of childhood asthma [32]. According to a cross-sectional study, serum 25 (OH) vitamin D level in asthmatic children was lower than in healthy children (12.88 + −1.79 ng/ml vs. 16.49 + −1.13 ng/ml) [30].

Asthmatic children spend more time indoors and are less exposed to sunlight which is necessary for vitamin D synthesis; therefore, this might be one of the reasons for lower levels of this vitamin [29, 30].

Vitamin D may play a role in asthma pathogenesis via different mechanisms, possibly through the development of the lungs, regulating the immune responses and remodeling of ASM [13].

Despite vitamin D deficiency being a global problem in all age groups, there are several limitations the recent reports. First, different methods have been used for measuring the vitamin D serum level which is difficult to do, and there has been large variations in results. Also, there are differences in the results of laboratories with the same methods [22]. Second, the size of samples in studies has been small; it is clear that for detecting association with more details, a larger sample size should be provided. The children with vitamin D levels of less than 20 ng/ml were not enough. Although there are limited studies to determine the effect of vitamin D supplementation on asthma exacerbation, most the children with vitamin D levels less than 30 ng/ml have levels greater than 20 ng/ml, so the result of studies could not be considered a general for most children with vitamin D insufficiency [30].

### 1.3. How Could the Low Vitamin D Level Affect Asthma?

#### 1.3.1. Respiratory System

Structural changes such as abnormally thickened epithelium with mucous gland hypertrophy, subepithelial membrane thickening, fibrosis with altered composition and deposition of extracellular matrix, angiogenesis, and increased ASM mass are the underlying pathophysiology of asthma [1]. Following these changes, narrowing of airways and contraction of the airway smooth muscle would occur [2, 34]. Airway remodeling begins in early childhood before the age of 3 years old [2].

Narrowing of airways is responsible for asthma symptoms [4], including prolonged coughing and recurrent wheezing [13]. Increased airway resistance [34] in response to multiple bronchoconstrictors, mediators, and neurotransmitters also lead to narrowing of the airway which can be reversed by bronchodilators [4].

Some studies have shown that lower serum 25(OH)D3 levels correlate with worse lung function [35], possibly due to the antiproliferation effect of vitamin D on airways which decelerates the cell cycle and halts hyperplasia of airway smooth muscle [1, 2]. Although low 25(OH)D3 levels were associated with increased ASM mass, the authors found no association between low 25(OH)D3 levels and airway inflammation despite an association with aeroallergen sensitization [35]. Vitamin D inhibits ASM proliferation by discontinuing the cell cycle, not through apoptosis [36], but by means of a reduction in calcitriol and subsequently serum and platelet derived growth factors [37].

On the other hand, 1,25(OH)2D3 can improve airway remodeling via downregulating the expression of matrix metalloproteinase-9 (MMP-9) [1, 2] synthesized by inflammatory cells. MMP-9 could digest IV_collagen [1]. It is suggested to be the key mediator of airway inflammation which can determine extracellular matrix (ECM) composition and immune cell infiltration in airway diseases [38]. Interestingly, tumor necrosis factor- (TNF-(*α* can increase MMP-9 activity [39]. VDR mediated the effect of calcitriol on TNF-*α*-induced MMP-9 activity [37], and its gene polymorphism is associated with asthma development [40]. Calcitriol increases the expression of TIMP-1 (tissue inhibitors of matrix metalloproteinases) in the presence of TNF-*α* and not independently [41]. TIMP-1 reduces MMP activity and has an antifibrotic roll in the lung [42].

Vitamin D can also reduce the expression of disintegrin metalloprotease-33 (ADAM-33) [2] which has a role in lung development and function; therefore, it can cause various features of asthma such as bronchial hyperresponsiveness, airway remodeling, lower lung functions, and accelerated lung function decline [2].

Active vitamin D receptors are expressed in ASM; therefore, 1,25-*α* dihydroxy vitamin D3 can play its role in the growth, morphogenesis, and survival of ASM via inducing the expression of 24-hydroxylase (CYP24A1) (this gene provided instructions for making 24_hydroxylase enzyme which controls the amount of active vitamin D available in the body) [2]. Banerjee et al. suggested that vitamin D could modulate the expression of chemokines in ASM, hence affecting airway reactivity [36]. Bosse ´ et al. showed that vitamin D can regulate the expression of many genes including genes involved in asthma predisposition and pathogenesis that can affect smooth muscle cell contraction and inflammation, as well as glucocorticoid and prostaglandin regulation. Vitamin D is also known for upregulating some genes with important roles in cellular movement, cellular growth and proliferation, and cellular death, hence leading up to asthma pathology by altering the airway remodeling process [43].

#### 1.3.2. Immune System

The immune system plays a key role in asthma pathogenesis. Th2 cells—one of the main compartments of cellular immunity—induce IgE and eosinophil production and are responsible for asthma pathogenesis via affecting the production of IL-4, IL-5, IL-9, and IL-13. Th1 cells, however, are involved in producing anti-inflammatory cytokines such as INF-*γ* which have a role in the early development of the infant immune system (possibly through TLR-2, TLR-4, and TLR-9) and establishing protection against asthma. Regulatory T-cells (Tregs), on the other hand, are inhibitors of Th2 and its cytokines and, therefore, are responsible for regulating immune system self-tolerance, preventing autoimmunity, and suppressing allergy reactions [8, 19].

Vitamin D hormone modulates immune response via biological process [44], and it could affect the development of the immune system [45].

Asthma is a type of disease which is driven by Th2 cells [46]. At low concentrations of vitamin D, CD4+ T cells express VDRs; then after activation of VDR, it increased 5-fold [46]. Vitamin D could suppress T cell proliferation and affect T cell maturation via shifting T helper cells, from a Th1 to a Th2 phenotype, reducing Th17 and increasing T regulatory cells; all these changes lead to increased production of anti-inflammatory cytokines (e.g., IL-10, IL-5, and IL-4) and decreased release of inflammatory cytokine, namely, IL-1, IL-12, and IL-18, TNF-*α*, and interferon gamma (IFN-*γ*) [47–50].

In addition to T helpers, dendritic cells (DCs) are also involved in the pathogenesis of asthma. DCs present antigen to T helper cells and activate them in an antigen-specific manner and subsequently induce naive CD4+ T cells to expand through an interaction between MHCII, T cell receptors (TCR), and costimulatory signaling molecules [51]. In other words, DCs promote the activation and proliferation of CD4+ T cells, as well as differentiation of Th cells during antigen presenting (Th cell differentiation is dependent on the strength and the duration of costimulatory and peptide-MHCII–TCR interactions) [52, 53].

Interestingly, vitamin D is capable of inhibiting the production of monocytes, such as IL-1, IL-6, IL-8, IL-12, and TNF-*α* [54]. Another function of vitamin D that demonstrates its key role in regulating the immune system is the ability of vitamin D to downregulate the expression of MHC class II molecules, costimulatory molecules, and IL-12 and inhibit DC differentiation and maturation by preserving the immature phenotype of dendritic cells [48]. If a mature DC presents antigen to the T cell, it will facilitate an immune response against that antigen, but when an immature DC presents the antigen, tolerance is more likely to occur [48].

Dendritic cells have well-known roles in activating Treg cells, as well. 1,25-(OH)2 D3 can induce the tolerogenic dendritic cell phenotype which produces IL-10. IL-0, in turn, promotes the expression of fork-head box P3 (FOXP3) Tregs. Upregulation of FOXP3 in Tregs activates T cells function and reverses steroid resistance [1, 2, 55].

**Figure 1:**
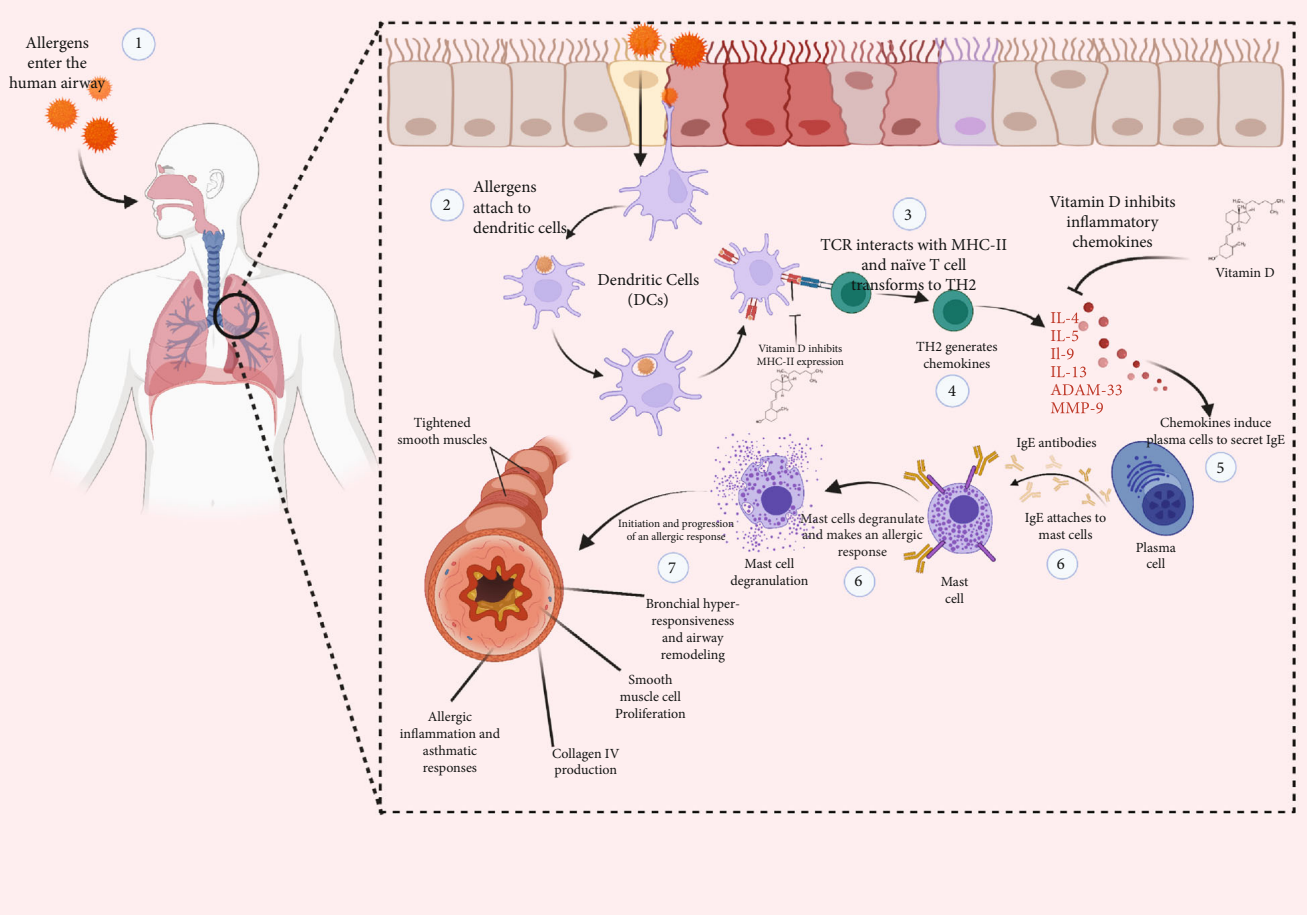
The role of vitamin D in immune mechanisms involved in asthma pathogenesis.

A summary of the underlying immune mechanisms is available in Figure 1.

### 1.4. Vitamin D Supplementation

Although lower vitamin D levels have been associated with more severe disease presentation, the role of vitamin D in decreasing the rate of first treatment failure, asthma exacerbations, and preventing asthma has not been proven yet [2, 13].

### 1.5. VIDA (Vitamin D Add-On) Therapy Enhances Corticosteroid Responsiveness

A number of studies have failed to find observable changes in the asthma status of patients who were treated with vitamin D (100,000 IU once then 4,000 IU/day for 28 weeks orally). The authors also reported no change in IgE levels and eosinophil count; yet, using monthly doses of vitamin D (60,000 IU per month for six months) reduced the rate of asthma exacerbations, the requirement for steroids and emergency room visits in asthmatic patients. Vitamin D supplementation significantly improved FEV_1_ (the amount of air you can force from your lungs in one second) in patients with mild to moderate persistent asthma after 24 weeks and is beneficial in improving the asthmatic respiratory infections treatment [2, 56–58].

The result of studies has shown that adding vitamin D3 supplementation to inhaled corticosteroids as an asthma treatment had no significant effect on the time to develop severe asthma and on morbidity from asthma [16, 17, 59, 60]. Vitamin D supplement did not significantly reduce the rate of first treatment failure, as well. Additionally, no effect on asthma control, airway function, asthma symptoms, quality of life, or airway inflammation was observed [61].

According to the results of a randomized clinical trial asthmatic people could taper inhaled corticosteroids to doses as little as 25% of the original dose, but there was a small difference in the absolute dose of corticosteroid [61]. Adding vitamin D supplementation to corticosteroid therapy did not cause less severe or less frequent colds than asthmatic people who did not receive vitamin D supplementation [62].

### 1.6. Prenatal Vitamin D Supplementation

The results of a randomized, double-blinded multicenter clinical trial showed that the 3-year incidence of asthma or recurrent wheezing in infants was 24.3% with 4400 IU/d and 30.4% with a 400 IU/d vitamin D supplement. This study demonstrated the effect of prenatal vitamin D (cholecalciferol) supplementation on preventing asthma or recurrent wheezing through 3 years of age yet the statistical difference was not significant; the answer remained unclear [63]. Another study showed that consuming vitamin D supplements during the prenatal period alone had no effect on asthma and recurrent wheeze among children who were at risk of asthma by the age of 6. The findings of this research showed that prenatal vitamin D supplementation could cause lower airway resistance and better spirometry lung function indexes but these effects were not certain [64]. A study that combined the analysis of the two trials reported a 26% reduction in the risk of asthma/recurrent wheeze in the offspring who had received vitamin D supplementation during the prenatal period cause. The risk was almost halved (46%) among women with 25(OH)D levels ≥ 30 ng/ml [65].

More researches are needed to prove these outcomes of VIDA effectiveness in asthmatic patients. Most of them support the involvement of vitamin D in lung growth and the development of the immune system and proinflammatory effects [1, 2, 13] .

## 2. Conclusion

An association between vitamin D deficiency and increased risk of childhood asthma has been known. This study is aimed at finding whether vitamin D supplementation could affect asthma treatment or not. The result of studies has shown that adding vitamin D3 supplementation to inhaled corticosteroids as an asthma treatment had no significant effect on the time to develop severe asthma and on asthma morbidity. Also, the effect of prenatal vitamin D (cholecalciferol) supplementation on preventing asthma or recurrent wheezing through 3 years of age yet the statistical difference was not significant. Further studies are recommended to evaluate the aspects of this vitamin effect on asthma development and management.
